# Revisiting the cerebral hemodynamics of awake, freely moving rats with repeated ketamine self-administration using a miniature photoacoustic imaging system

**DOI:** 10.1117/1.NPh.9.4.045003

**Published:** 2022-11-01

**Authors:** Yuhling Wang, Chia-Hua Tsai, Tsung-Sheng Chu, Yun-Ting Hung, Mei-Yi Lee, Hwei-Hsien Chen, Li-Tzong Chen, Tzong-Rong Ger, Yung-Hsuan Wang, Nai-Jung Chiang, Lun-De Liao

**Affiliations:** a National Health Research Institutes, Institute of Biomedical Engineering and Nanomedicine, Zhunan Town, Miaoli County, Taiwan; b Chung Yuan Christian University, Department of Biomedical Engineering, Taoyuan City, Taiwan; c National Health Research Institutes, Center for Neuropsychiatric Research, Zhunan Town, Miaoli County, Taiwan; d Kaohsiung Medical University, Kaohsiung Medical University Hospital, Kaohsiung City, Taiwan; e National Health Research Institutes, National Institute of Cancer Research, Zhunan Town, Miaoli County, Taiwan; f Taipei Veterans General Hospital, Department of Oncology, Taipei City, Taiwan

**Keywords:** freely moving animals, ketamine addiction, in vivo imaging, fiber bundle-based illumination, hemoglobin oxygenation saturation

## Abstract

**Significance:**

Revealing the dynamic associations between brain functions and behaviors is a significant challenge in neurotechnology, especially for awake subjects. Imaging cerebral hemodynamics in awake animal models is important because the collected data more realistically reflect human disease states.

**Aim:**

We previously reported a miniature head-mounted scanning photoacoustic imaging (hmPAI) system. In the present study, we utilized this system to investigate the effects of ketamine on the cerebral hemodynamics of normal rats and rats subjected to prolonged ketamine self-administration.

**Approach:**

The cortical superior sagittal sinus (SSS) was continuously monitored. The full-width at half-maximum (FWHM) of the photoacoustic (PA) A-line signal was used as an indicator of the SSS diameter, and the number of pixels in PA B-scan images was used to investigate changes in the cerebral blood volume (CBV).

**Results:**

We observed a significantly higher FWHM (blood vessel diameter) and CBV in normal rats injected with ketamine than in normal rats injected with saline. For rats subjected to prolonged ketamine self-administration, no significant changes in either the blood vessel diameter or CBV were observed.

**Conclusions:**

The lack of significant change in prolonged ketamine-exposed rats was potentially due to an increased ketamine tolerance. Our device can reliably detect changes in the dilation of cortical blood vessels and the CBV. This study validates the utility of the developed hmPAI system in an awake, freely moving rat model for behavioral, cognitive, and preclinical cerebral disease studies.

## 1 Introduction

One of the most significant challenges in modern neuroscience is thoroughly understanding the interactions between brain structures and functions.^1^ To develop human medicines more quickly and with more confidence, it is important to devise innovative imaging techniques that allow scientists to observe active brains. With the development of new brain imaging methods, it has become possible to track disease activity, and the measurement sensitivity of cerebral characteristics has improved beyond current clinical observations. Imaging has been used to directly visualize molecular interactions to better understand cerebral responses in patients, which can help in determining the optimal dose of experimental medicines. With the enhanced information provided by imaging, the effects of new medicines can be predicted by revealing the detailed outcomes of the medicine on brain functions relevant to the target diseases.

Existing imaging techniques that allow scientists to visualize brain functions rely primarily on two main approaches: (1) magnetic resonance imaging (MRI) and (2) positron emission tomography (PET).^1^ MRI uses benign magnetic fields and radio waves to visualize cerebral structures and physiology. MRI is one of the main techniques used to map the brains of humans and animals due to its noninvasiveness, high contrast in soft tissue, and multiple readouts. In addition, PET uses radioactive tracers at safe doses to perform molecular imaging in humans and animals. These two techniques can accelerate the process of drug development.

However, alternative approaches are needed to closely examine dynamic interactions between vascular and neural components during the drug discovery process. For example, electrophysiological recordings can be combined with different optical absorption-sensitive imaging methods, such as near-infrared spectroscopy, optical intrinsic signal imaging, and photoacoustic (PA) imaging.^1^ Optical scattering-sensitive imaging methods, including optical coherence tomography (OCT),^2^^,^^3^ laser speckle contrast imaging (LSCI),^4^ and diffuse correlation spectroscopy,^5^ can also be used. Fluorescence-sensitive techniques using exogenous dyes have also been extensively explored, including two-photon microscopy (TPM), wide-field imaging, and voltage-sensitive dye imaging.^6^^,^^7^ When these optical imaging techniques are compared, PA imaging can penetrate tissues at greater depths and has higher spatial resolution than other optical absorption-based techniques.^8^^–^^10^

This study utilizes a miniature head-mounted scanning photoacoustic imaging (hmPAI) system to study the effects of ketamine-induced cortical hemodynamic responses in awake, freely moving rats. Four experimental groups were designed and analyzed in this study. In the first group, hemodynamic changes in normal rats injected with saline were investigated. In the second group, hemodynamic changes in the brains of normal rats after ketamine injection were investigated and compared with the first group of rats injected with saline. In the third group, trained ketamine-addicted rats were injected with saline, and hemodynamic changes were monitored through the same system. Finally, in the fourth group, ketamine-addicted rats were injected with ketamine, and the hemodynamic effects of acute ketamine injection on the cerebral blood vessels of addicted rats were observed. The hmPAI system was applied to study the effects of ketamine-induced cortical hemodynamic responses in awake, freely moving rats. For these four experimental groups, the PA signals were obtained as A-line and B-scan signals, and the changes in the received PA signals were used to determine whether ketamine affected the cerebral blood vessels and/or cerebral blood volume (CBV). The hmPAI system has dimensions of only 50  mm×64  mm×48  mm, and its total mass is ∼58.7  g, excluding any cables. Our previous studies showed that anesthesia can have a confounding effect on neural activity. When awake and anesthetized animals were compared, several differences were detected, including how neurotransmitters bind to receptors^11^ and changes in the relative cerebral blood volume and other responses to stimuli.^12^ In addition, not all brain regions were equally affected by anesthesia,^13^ providing an additional confounding factor. Here, our hmPAI system allowed us to study the effect of ketamine on awake and freely moving rats without the confounding effects of anesthesia. We first used PA B-scan and C-scan imaging to assess the real-time cortical hemodynamic changes induced by ketamine in normal and ketamine-addicted rats. Next, we compared the real-time cortical hemodynamic changes with cortical hemodynamic changes induced in the same brain regions by ketamine in the ketamine-addicted rat model. Overall, the goal of this compact hmPAI system is to meet the diverse needs of neuroscientists performing preclinical cerebral studies.

## 2 Materials and Methods

### 2.1 Miniature hmPAI System

**Fig. 1:**
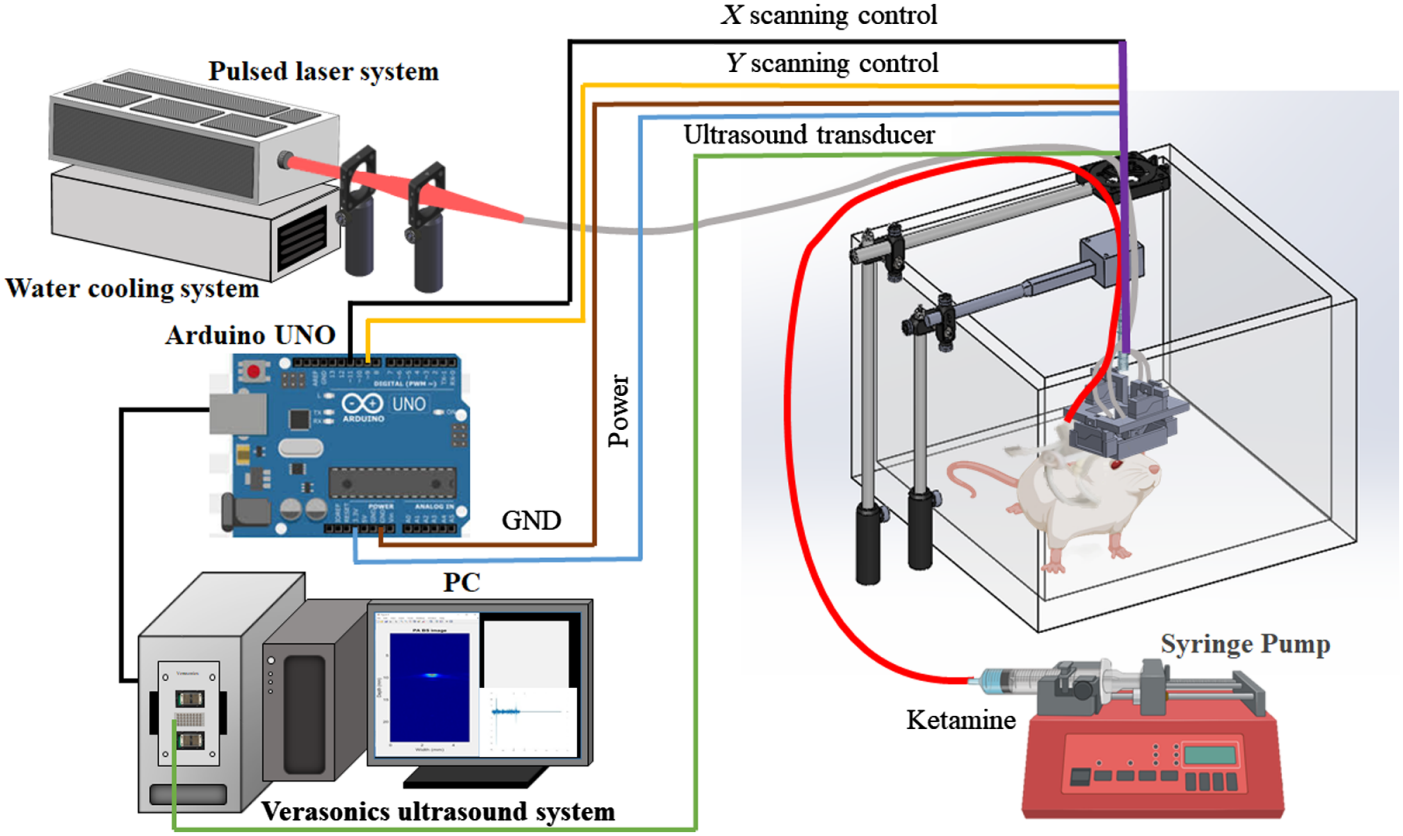
Schematic diagram of the developed hmPAI system with the real-time ketamine-treated model. Saline and ketamine were quantitatively injected through a syringe pump and evaluated with the hmPAI system. The hmPAI system was controlled by Arduino UNO, and the Verasonics system was used to collect signals. A passive weight support system was used to reduce the burden of the hmPAI system on the rats. The weight support system had two brackets: one to fix the optical fiber to enable irradiation with the pulsed laser and one to reduce the weight of the device. The rats used in the experiment wore jackets that were connected to the tube and the jugular vein of the rats; thus, we used the injection pump to inject normal saline and ketamine regularly and quantitatively to facilitate the experiments. Our device can be used to determine whether ketamine affects the cerebral blood vessels of rats. A jacket was placed on the animal, and we connected the jacket and blood vessels with a tube through a surgery in advance. An injection pump was used to inject ketamine and saline at a fixed rate during the experiment.

Figure 1 shows a schematic of the developed hmPAI system, including the scanning control, laser illumination, PA/ultrasound (US) signal acquisition, and experimental setup. The multichannel Verasonics high-frequency US platform (Vantage 128, Verasonics Inc., Kirkland, Washington) was employed for dual-modality imaging (PA imaging and US imaging). The hmPAI system was fully controlled by a custom graphical user interface developed in MATLAB^®^ (R2007a, MathWorks Inc., Massachusetts, Boston). To operate the system in US or PA mode, a trigger was used to synchronize the laser excitation and data acquisition. A wideband 48-MHz ultrasonic transducer with a large numerical aperture was used to efficiently collect the PA signals generated by cortical blood vessels. This transducer had a −6-dB fractional bandwidth of 57.5%, a focal length of 9 mm, and a 6-mm active element.^14^ The excitation laser was a compact Nd:YAG laser system with an integrated tunable optical parametric oscillator (OPO) system (SpitLight 600 OPO, InnoLas Laser GmbH, Krailling, Bavaria, Germany). The OPO generates ∼7  ns pulses at a 20-Hz repetition rate with tunable wavelengths ranging from 680 to 2400 nm.^14^ A fiber bundle was used to transmit laser light to the tissue surface through four output ends. The fiber output ends were positioned to illuminate the tissue in an approximately ring-shaped region to achieve dark-field illumination.^15^

The detailed design of the miniature head-mounted holder for the hmPAI system is shown on the right side of Fig. 1. An Arduino UNO controller (Arduino Corp., Ivrea, Italy) provided the power supply and scanning control for the four linear servo motors. Two Y-axis motors (versus-19, Solarbotics Ltd., Canada) and two X-axis motors (versus-19, Solarbotics Ltd., Canada) were controlled by digital signals for y- and x-direction scanning, respectively. The minimum step size for the selected linear motor was ∼0.12  mm (based on the experimental results). The hmPAI system was developed to provide A-scans, B-scans (i.e., 2D images with one axis being the lateral scanning distance and the other axis being the imaging depth), and C-scans (i.e., 3D images) of the region of interest (ROI). A 3D-printed Y-axis holder with two slots was designed to hold the two linear motors, and a hollow sink in the center part of the holder was designed as a water tank to ensure efficient acoustic coupling during scanning. A 3D-printed X-axis holder with two slots was designed to hold the X-axis linear motors. The 3D-printed holder for the transducer was designed with four slots to hold the four output ends of the fiber bundle at the appropriate light emission angles and a single slot to tightly hold the selected ultrasound transducer. The PA probe (including the removable fiber bundle-based illumination system, one US transducer, and a customized jacket) was attached to the scanning stage with a 3D-printed holder. The X-axis and Y-axis holders were designed using the computer-aided design software package SolidWorks 2015 (Dassault Systemes SA., Massachusetts) and fabricated using a 3D printer (Shuffle 4k, Phrozen, Inc., Hsinchu, Taiwan) with an accuracy of 0.03 mm (ABS Like). The incident energy density at the imaging sample surface was estimated to be ∼14 to $20\;\mathrm{mJ}/{\mathrm{cm}}^{2}$, which did not exceed the American National Standards Institute safety limit ($20\;\mathrm{mJ}/{\mathrm{cm}}^{2}$).

### 2.2 Experimental Animals

In total, six male SD rats (National Laboratory Animal Center, Taiwan) weighing between 250 and 350 g were used in the cortical blood vessel imaging experiments, with PA A-line and B-scan imaging performed at the same bregma locations. Three normal rats were injected with saline or ketamine (i.e., group 1 and group 2) to compare the hemodynamics of in the brain before and after injection. Additionally, three ketamine-addicted rats were injected with saline or ketamine (i.e., group 3 and group 4), and the hemodynamics of the brain were compared before and after injection. All animals were housed in a controlled room (temperature 24±1°C, relative humidity 50% to 60%) on a 12/12 h light/dark cycle (light phase beginning at 7 am) with free access to food and water.^16^ All experimental procedures, animal care protocols, and protocols requiring ethical oversight were performed following the guidelines approved by the Institutional Animal Care and Use Committee of the National Health Research Institute (approved protocol number: NHRI-IACUC-107100-A). The animals were initially anesthetized with 3% isoflurane (Bowlin Biotech Corp., Taoyuan, Taiwan). The anesthetized rats were mounted on a custom acrylic stereotaxic head holder,^17^ and the skin and muscle were removed from the skull to expose the bregma, which was used as a landmark.^18^ The anteroposterior distance between the bregma and the interaural line was directly measured;^19^ the bregma was found to be located at a distance of 9.3±0.12  mm (mean ± standard deviation) from the interaural line.^16^ Furthermore, a craniotomy was performed for each animal, and a bilateral cranial window of approximately 8 (horizontal) × 6 (vertical) mm was created using a high-speed drill.

### 2.3 Experimental Protocol and Timeline for the Ketamine Addiction Rat Model

**Fig. 2:**
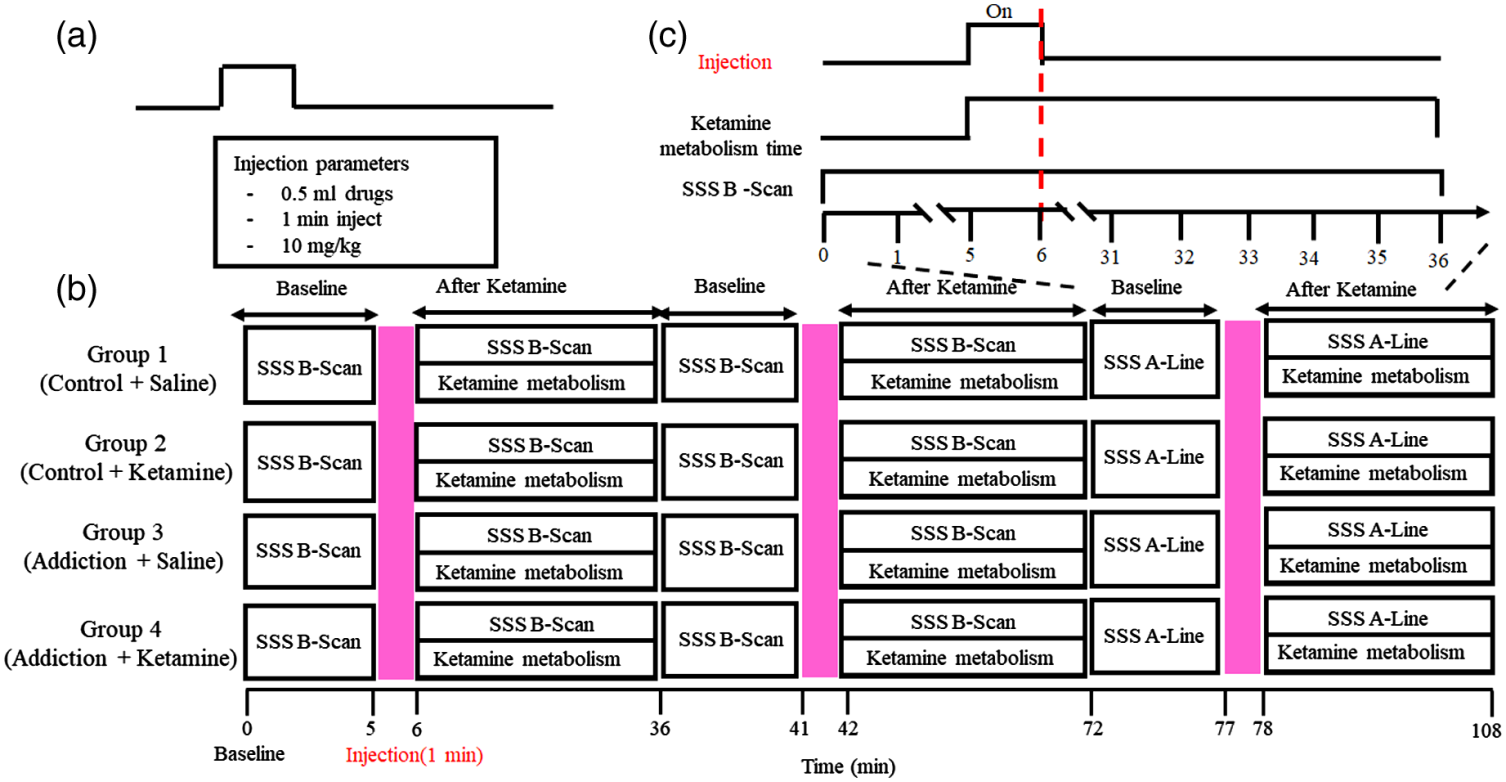
Experimental protocol and timeline for the hmPAI system. (a) The saline or ketamine injection of 0.5 ml was given over 1 min at a consistent dose of 10 mg per kg. (b) The purpose of group 1 was to observe the effect of saline on cerebral blood vessels in rats. First, we collected the vascular signal 5 min before the saline injection; then, we injected saline for one minute and observed the changes in the cerebral blood vessels in the following 30 min. The results of groups 1 and 2 were compared. The saline was replaced with ketamine to compare the effects of ketamine on cerebral blood vessels. In group 3, we replaced normal rats with ketamine-addicted rats, injected saline with the same experimental technique, and observed whether the blood vessels changed significantly. Group 4 also used ketamine-addicted rats. This group was injected with ketamine to determine whether ketamine had the same effect on cerebral blood vessels in addicted and normal rats. The PA signal was recorded before ketamine injection for 5 min to acquire a baseline; then, the PA signal was recorded for 30 min after ketamine metabolism. (c) The PA signals were acquired using the developed hmPAI system and a block design paradigm. The task began in the baseline state for 5 min. Then, a constant concentration of 10 mg per 1 kg was injected for 1 min during the ON state, which was followed by a recovery period of 30 min. During this period, the PA signals were continuously collected to observe the cerebrovascular changes induced by saline and ketamine.

The ketamine experimental protocol and timeline for the hmPAI system are shown in Fig. 2. A ketamine injection of 0.5 ml was given over 1 min at a consistent dose of 10 mg per kg [Fig. 2(a)]. Figure 2(b) shows the four experimental groups. Group 1 consisted of normal rats injected with saline; group 2 consisted of normal rats injected with ketamine; group 3 consisted of ketamine-addicted rats injected with saline; and group 4 consisted of ketamine-addicted rats injected with ketamine. The experimental protocol is shown in Fig. 2(b). The baseline PA signal was recorded for 5 min (before saline or ketamine injection); then, the PA signal was recorded for 30 min after injection in the four groups [i.e., the normal rats injected with saline (group 1), the normal rats injected with ketamine (group 2), the addicted rats injected with saline (group 4) and the addicted rats injected with ketamine (group 4)]. The PA signals were acquired with a block design paradigm, as shown in Fig. 2(c). The task began in the baseline state for 5 min. Then, a constant concentration of 10 mg per 1 kg was injected for 1 min during the “ON” state, which was followed by a recovery period of 30 min.

### 2.4 Ketamine Self-Administration Rat Model

The rats in groups 3 and 4 were trained to self-administer ketamine in a substance abuse animal model. In this model, the rats were pretrained to press a lever using food pellets. The rats were food-restricted (5  g/day) for 48 h prior to food training. During the 1 h training session, the animals were trained to press a lever to receive a single food pellet (45 mg; BioServe Biotechnologies, Ltd., Beltsville, Maryland) at a fixed ratio of 1. Only one lever was extended into the operant testing chamber during the initial food training period. Animals took 3 to 4 days to meet the criteria (defined as earning 100 food pellets within the 1 h session for three consecutive days).

Next, surgery was performed to implant tubing for ketamine delivery. Rats received intravenous catheterization surgery under isoflurane (2% v/v) anesthesia at least 3 days after the completion of food pretraining. The external jugular vein was implanted with Silastic^®^ tubing (ID=0.51  mm; OD=0.94  mm; Dow Corning, Midland, Michigan). The other end of the tubing was connected to an injection port in a harness (Instech Laboratories, Plymouth Meeting, Pennsylvania). The catheters were flushed daily with a mixed solution of Baytril antibiotic (2.5%; Bayer AG, Leverkusen, Germany) and heparinized saline (50  IU/ml) to preserve catheter patency. The rats were fed ad libitum for 7 days following surgery, after which the rats were given 15 g of food immediately after each daily drug self-administration session.

After surgery, the rats were trained to self-administer ketamine. All self-administration sessions were conducted in operant chambers (32×25×34  cm, Med Associates, Fairfax, Vermont) that were housed in sound-attenuating cubicles with a ventilation fan and linked to a computerized data collection program. Each chamber included two retractable levers and yellow stimulus lights above each lever.

The rats participated in 2 h daily training sessions. Each time the rat pressed the active lever, the syringe pump delivered ketamine (0.5  mg/kg/infusion) from FR1 to FR2. Each ketamine infusion (4 s) was followed by a 20 s timeout period (TO20), during which additional active lever presses were recorded but produced no programmed responses. Each ketamine infusion was accompanied by illumination of the stimulus light for 20 s. The animals received a 0.1 ml infusion of heparinized saline (33.3  U/ml) after each self-administration session. The self-administration training sessions were conducted until the response patterns of the rats stabilized (i.e., the number of active lever presses per 2 h session varied <15% across two consecutive sessions).

### 2.5 Jugular Vein Surgical Procedure for the Ketamine Addiction Experiment

**Fig. 3:**
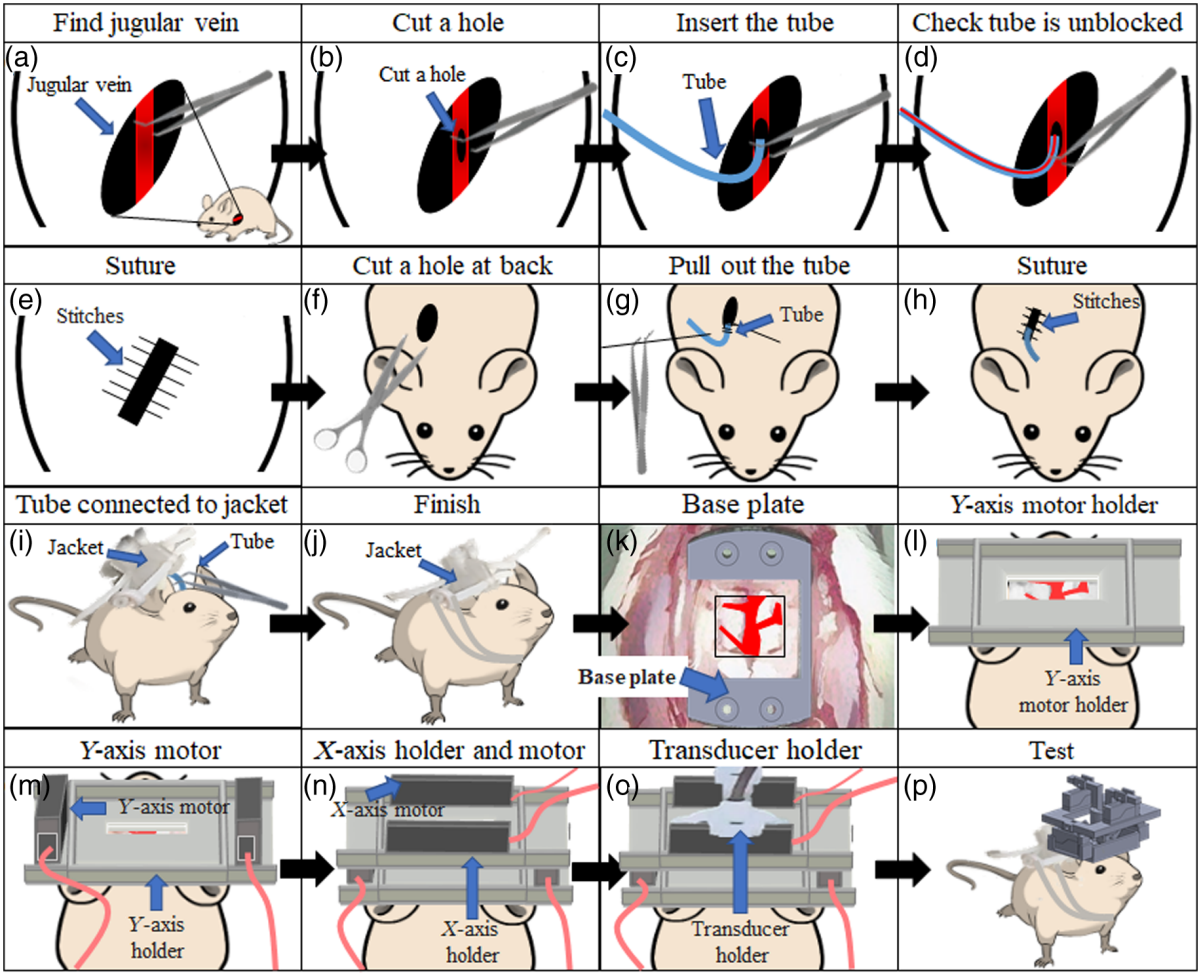
Schematic diagram of the surgical procedure to connect the jugular vein with the hmPAI system. (a) A hole was made in the chest, skin, and muscle to find the jugular vein. (b) A small incision was cut in the jugular vein (c). The tube was inserted into the incision in the jugular vein. (d) The tube was verified to be unblocked (e). The tube was closed with a surgical suture. (f) A hole was cut in the back. (g) The other side of the tube was pushed out the hole in the back (h). The wound on the back was sutured. (i) The tube was connected to the jacket. (j) The surgical procedure with the jugular vein was completed. (k) Four screws were used to lock the base plate. (l) The Y-axis motor holder was placed in the base plate, and glue was used to secure the connection. (m) The two Y-axis linear motors were placed in the Y-axis motor holder, and four screws were used to secure the motor in the holder. (n) Two screws were used to secure the X-axis holder and Y-axis motor. (o) The transducer was secured to the X-axis motor using two screws. (p) The PA signal in the surgical area was collected.

First, surgery was performed to connect the jugular vein to the jacket for ketamine and saline injections during experiments. Holes were cut in the chest, skin, and muscle to expose the jugular vein [Fig. 3(a)]. Next, a small incision was cut in the jugular vein [Fig. 3(b)]. A tube was inserted into the small incision in the jugular vein [Fig. 3(c)] and verified to be patent, as shown in [Fig. 3(d)]. The inserted tube was connected to the jugular vein with a surgical suture [Fig. 3(e)]. Next, a hole was cut in the back of the animal [Fig. 3(f)]. The other end of the tube was pulled through this hole in the back [Figs. 3(g) and 3(h)] and connected to the jacket [Fig. 3(i)]. All wounds were closed with surgical sutures [Fig. 3(j)], completing the operation to connect the jacket and the jugular vein.

### 2.6 Surgical Procedure for the Developed hmPAI System

After the craniotomy, four holes were drilled, and four screws were used to secure the base plate [Fig. 3(k)]. The Y-axis motor holder was placed on the base plate and secured [Fig. 3(l)] with an angled slot mechanism. The angled slot mechanism and glue were used to stably affix the system to ensure that motion artifacts were minimized. Next, two Y-axis linear motors were placed in the Y-axis motor holder, and four screws were used to secure the motors in the holder, as shown in Fig. 3(m). Then, two screws were used to secure the X-axis holder with the Y-axis motor, and four screws were placed in the X-axis motor holder [Fig. 3(n)]. Finally, the transducer was secured to the X-axis motor using two screws [Fig. 3(o)]. After the above steps, analgesics were given. The experimental rat recovered for 1 h; then, the rat was moved to the box to start the test. The cerebral blood vessels were monitored during the experiment through the hmPAI system placed on the top of the rat brain, as shown in [Fig. 3(p)].

### 2.7 Imaging of Cortical Hemodynamic Changes in the Superior Sagittal Sinus

After the PA B-scan, the rat brain vasculature in the region of the bregma was imaged for 5 min. Next, ketamine was injected through the jacket. A 0.5-ml volume of ketamine was infused over 1 min at a consistent dose of 10 mg per kg. A laser excitation wavelength of 800 nm was used to acquire the PA images. Blood is predominantly an absorber at the selected wavelength, and this strong optical absorption guarantees that the detected PA signals mainly originate from blood vessels.

We monitored changes in the *in vivo* ${\mathrm{PA}}_{800}$ A-line signal as a measure of the cortical superior sagittal sinus (SSS) diameter at the position of the bregma in normal and addicted rats. The A-line PA signal along the axial direction was normalized and fitted to a Gaussian distribution function. The axial diameter was calculated as the full-width at half-maximum (FWHM) of each Gaussian function.^20^ The A-line PA signal and FWHM of the PA A-line signal changed over time in both normal and addicted rats, and these changes were tracked. To monitor changes in the CBV, an ROI was created around the blood vessel in *in vivo* ${\mathrm{PA}}_{800}$ B-scans of the cortical SSS at the position of the bregma in normal and addicted rats. The number of pixels in the ROI were quantified to determine the CBV due to the use of the 800-nm wavelength light.^21^

## 3 Results

Despite the miniaturized size, the proposed hmPAI system provided good spatial resolution (i.e., an axial resolution of 0.225 mm^22^) and successfully captured rapid cerebral hemodynamic changes (2  lines/s for PA A-line signals) in awake and freely moving rats. Previously, we conducted *ex vivo* phantom tests and *in vivo* experiments to demonstrate the capability of the hmPAI system for small animal PA imaging.^15^ In this study, we performed imaging tests to detect changes in the cortical blood vessel diameter and CBV in awake, freely moving rats using light with a wavelength of 800 nm. The diameter and CBV of the selected blood vessels changed, and a significantly larger amplitude was observed in awake, freely moving rats injected with ketamine than in rats injected with saline in the nonaddicted model. Additionally, based on the PA C-scan data, the regional hemodynamics in ketamine-addicted and normal rats in awake, freely moving states were significantly different at the same bregma position.

### 3.1 Effects of Ketamine on Blood Vessel Diameter Changes

**Fig. 4:**
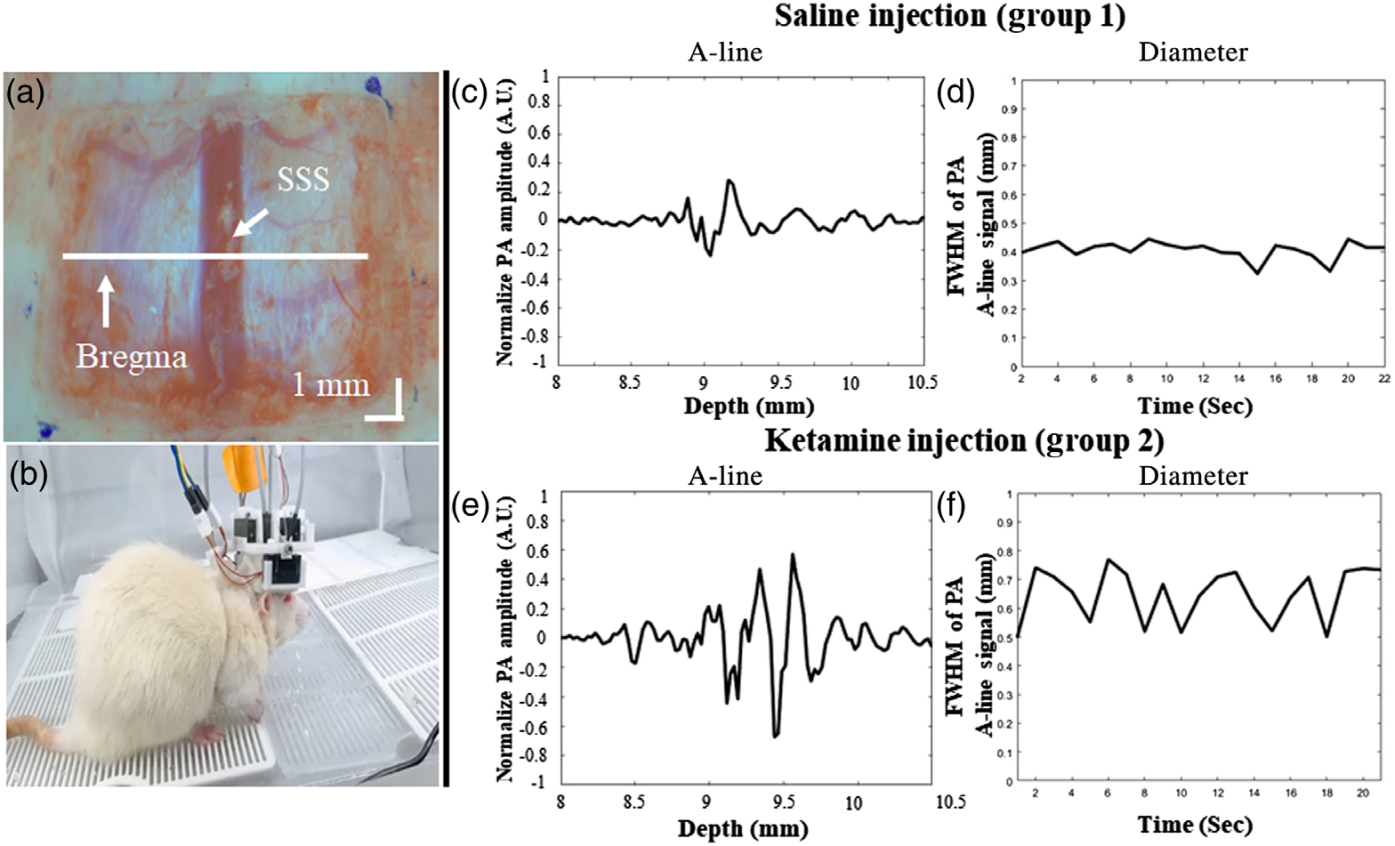
*In vivo* ${\mathrm{PA}}_{800}$ FWHM of the A-line signal of brain SSS changes at the position of the bregma in saline-injected and ketamine-injected rats. (a) Photograph of the rat brain after craniotomy for hmPAI system monitoring. (b) Photographs show the freely moving experimental rats while wearing the hmPAI system (Video 1). (c) ${\mathrm{PA}}_{800}$ A-line signal of cortical SSS at the bregma after saline injection (Video 1). (d) The diameters of cortical SSS blood vessels in the brains of saline-injected rats were monitored in real time with 800 nm excitation (Video 1); the diameters ranged from 0.31 to 0.73 mm. (e) ${\mathrm{PA}}_{800}$ A-line signal of cortical SSS blood vessel diameter at the bregma in ketamine-injected rats (Video 1). (f) The diameters of cortical SSS blood vessels in the brains of ketamine-injected rats were monitored in real time with 800 nm excitation (Video 1); the diameters ranged from 0.34 to 1.22 mm (Video 1, MP4, 59.4 MB [URL: https://doi.org/10.1117/1.NPh.9.4.045003.s1).

**Fig. 5:**
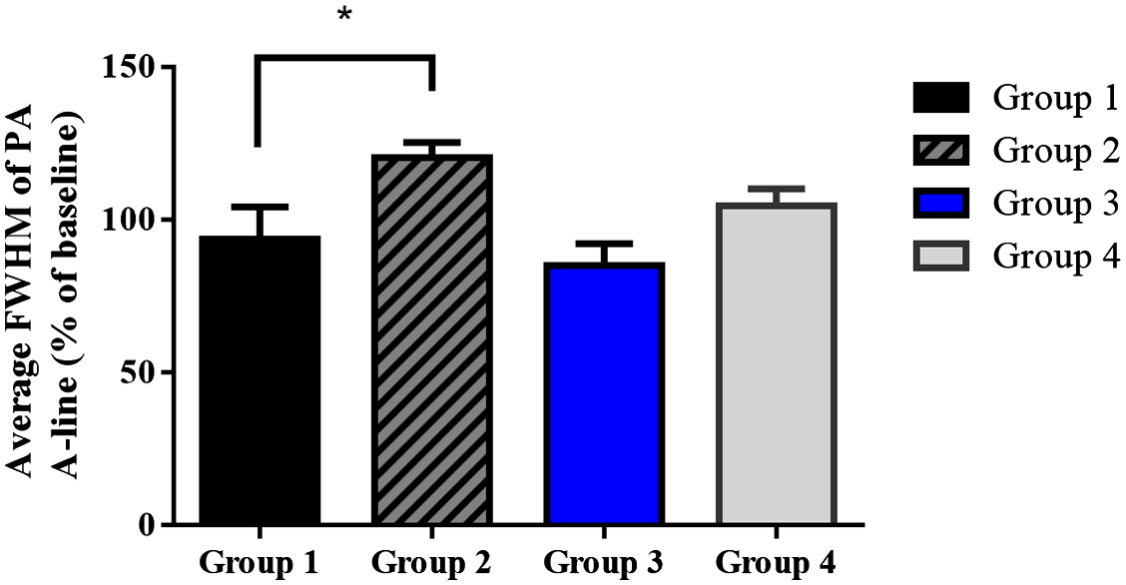
FWHM of the PA A-line signal baseline in groups 1, 2, and 3. The black, gray, and blue bars indicate the FWHM of the A-line amplitude as a percentage of the baseline. The error bars represent the SD. * p<0.05 (paired t-test, n=3). Normal rats injected with saline or ketamine were compared, and the change in the blood vessels in group 2 after ketamine injection was 93.71±10.47%, whereas the change in the blood vessels in group 1 after saline injection was 120.42±4.89%, with a significant difference of p<0.05 (p=0.0365) (paired t-test, n=3 each for group 1 and group 2). Prolonged ketamine self-administration rats that were injected with ketamine or saline in groups 3 and 4 showed no significant difference, with p>0.05 (p=0.1123) (paired t-test, n=3 for both group 3 and group 4).

Figure 4 reports the *in vivo* changes in the ${\mathrm{PA}}_{800}$ A-line signal as a measure of the cortical SSS blood vessel diameter at the position of the bregma following saline and ketamine injection in both normal and ketamine-addicted rats. After the craniotomy [Fig. 4(a)] and device placement for hmPAI monitoring, the experimental rats moved freely while equipped with the hmPAI system, as reflected by the photographs in Fig. 4(b) (Video 1). The A-line PA signals along the axial direction were normalized and fitted to Gaussian distribution functions. The axial diameter was calculated as the FWHM of each Gaussian function. Figures 4(c) and 4(d) show the changes in the A-line PA signal and FWHM of the A-line signal at various time points in the saline-treated rats (Video 1). Figure 4(d) shows that the diameter of the blood vessels ranged from 0.31 to 0.73 mm. Figures 4(e) and 4(f) show the changes in the A-line PA signal and FWHM of the A-line signal at various time points in the ketamine-treated rats (Video 1). Figure 4(f) shows the changes in the A-line signal, which indicates that the blood vessel size ranged from 0.34 to 1.22 mm. The changes in the cerebral blood vessel dilation after ketamine infusion in normal and addicted rats are shown in Fig. 5. The statistical analysis results show that the changes in the FWHM of the A-line signal as percentages of the baseline in groups 1 and 2 were 93.71±10.47% and 120.42±4.89%, respectively [p<0.05 (p=0.0365), paired t-test; n=3 for both group 1 and group 2]. These results show that the cortical blood vessels expand significantly after ketamine injection, which suggests that the brain needs an increased blood supply after ketamine injection, which causes the blood vessels to expand. Furthermore, the changes in the FWHM of the A-line signal as percentages of the baseline in groups 3 and 4 were 85.03±7.11% and 104.62±5.43%, respectively [p>0.05 (p=0.1123), paired t-test, n=3 for both group 3 and group 4]. These results show that in addicted rats, the effect of ketamine injection may be influenced by habituation to ketamine, as the blood vessels in the brain did not expand noticeably, proving that our device can reliably detect changes in the dilation of cortical blood vessels.

### 3.2 Effects of Ketamine on CBV Changes

According to optical absorption spectra information found in the literature, the wavelength of 800 nm is sensitive to changes in the CBV.^23^^,^^24^ Furthermore, PA imaging at 800 nm contains various signals that are generated by different types of tissues, such as changes in the CBV.^22^^,^^25^ Therefore, the PA images acquired at 800 nm were used to detect changes in the CBV. Thus, independent measurements of changes in the CBV can be achieved by the developed wearable scanning PA imaging system with PA imaging at 800 nm being used to monitor changes in the CBV.

**Fig. 6:**
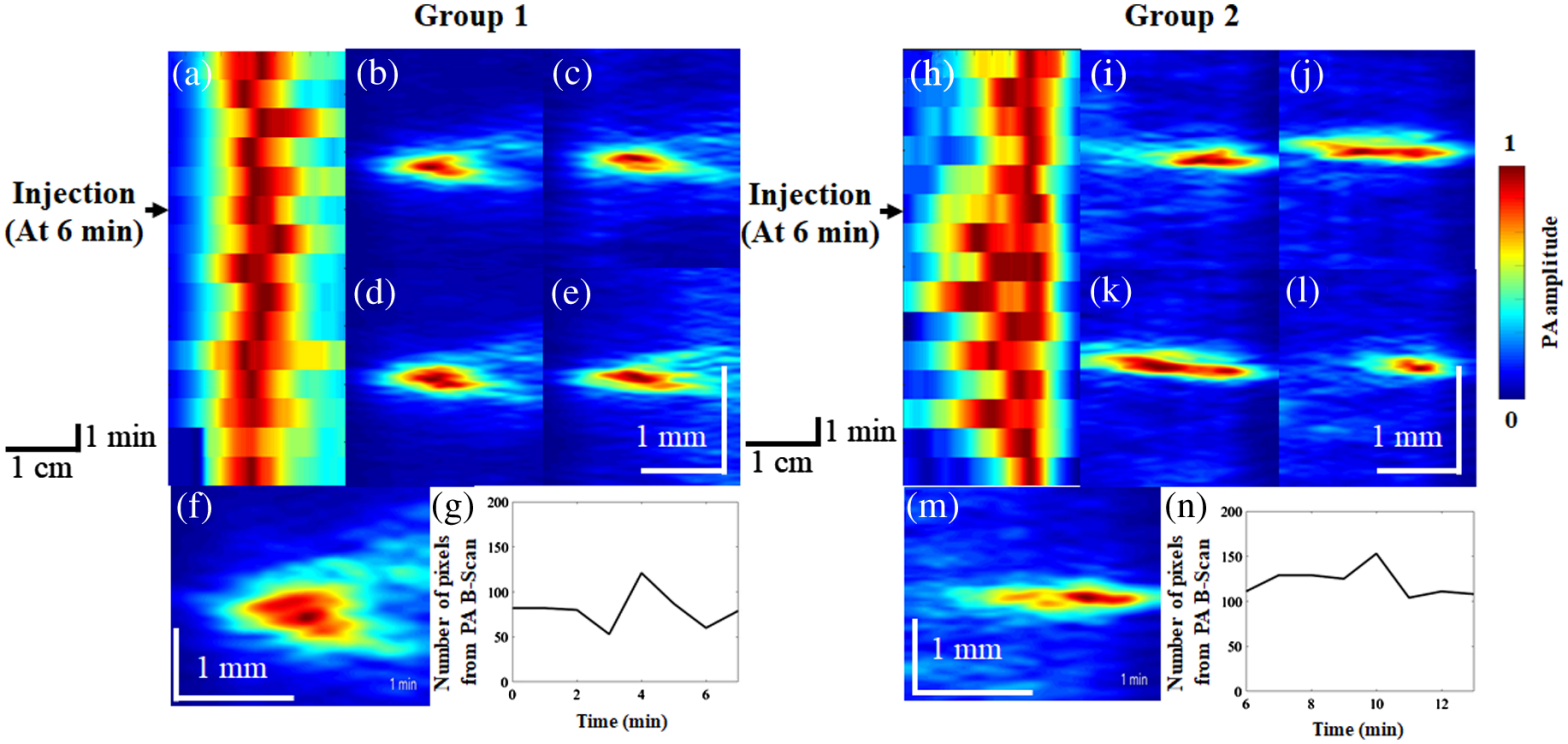
B-scan monitoring of vasoconstriction and vasodilation induced by saline and ketamine injection. The injection always began at the sixth minute and took place over the course of 1 min. Quantitative saline and ketamine were injected at the same rate through the injection pump. (a) The PA maximum amplitude projection (MAP) image of the time course of the vessel diameter changes in saline-injected rats. (b)–(e) Cross-sectional vessel images at different time points after saline injection. (f) The PA B-scan images of saline-injected rats were combined in a video to monitor vessel changes (Video 2). (g) The vessel diameter changes were monitored throughout the video (Video 2), with the number of pixels in the image ranging from 52 to 123. (h) The PA MAP image of the time course of the vessel diameter changes in ketamine-injected rats. (i)–(l) Cross-sectional vessel images at different time points after ketamine injection. (m) The PA B-scan images of ketamine-injected rats were combined in a video to monitor vessel changes (Video 2). (n) The vessel diameter changes were monitored throughout the video (Video 2), with the number of pixels in the image ranging from 58 to 162 (Video 2, MP4, 4.40 MB [URL: https://doi.org/10.1117/1.NPh.9.4.045003.s2).

Figure 6 reports B-scan monitoring of vasoconstriction and vasodilation in groups 1 and 2. Figure 6(a) shows the time course of blood vessel diameter changes in rats in group 1. Figures 6(b)–6(e) show cross-sections of blood vessels of rats in group 1 at different time points. Figure 6(f) shows PA B-scan images that were combined in a video to monitor blood vessel changes in group 1 (Video 2). The number of pixels in PA B-scan images of rats in group 1 at various time points is shown in Fig. 6(g) (Video 2), with the number of pixels ranging from 52 to 123. Figure 6(h) shows the time course of blood vessel changes in rats in group 2. Figures 6(i)–6(l) present cross-sections of blood vessels of rats in group 2 at different time points. Figure 6(m) shows PA B-scan images that were combined in a video to monitor blood vessel changes in group 2 (Video 2). The number of pixels in PA B-scan images of rats in group 2 at various time points is shown in Fig. 6(n) (Video 2), with the number of pixels ranging from 58 to 162.

**Fig. 7:**
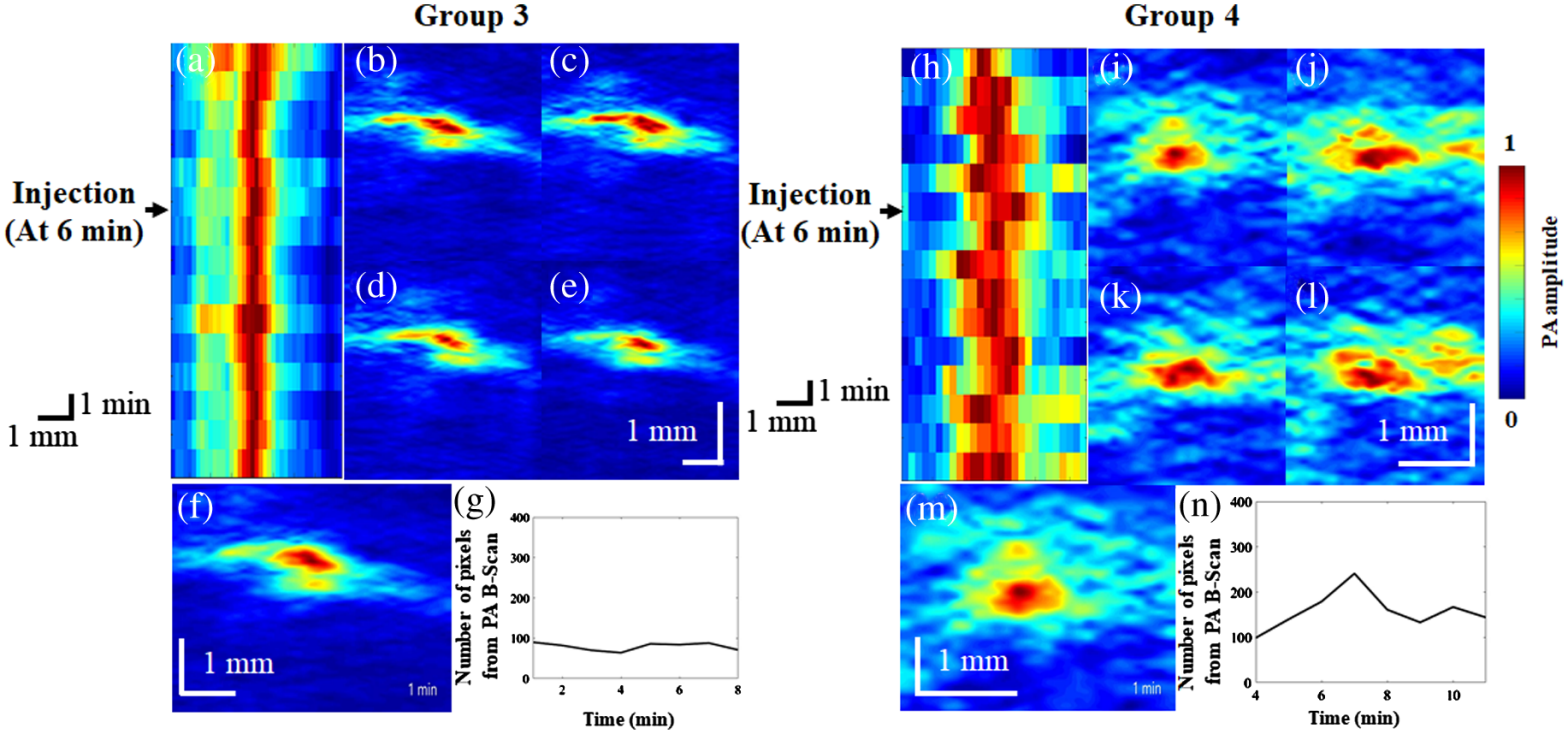
PA B-scan monitoring of vasoconstriction and vasodilation induced by saline and ketamine injection. (a) The PA MAP image of the time course of vessel diameter changes in saline-injected rats. (b)–(e) Cross-sectional vessel images at different time points after saline injection. (f) The PA B-scan images of saline-injected rats were combined in a video to monitor vessel changes (Video 3). (g) The vessel diameter changes were monitored throughout the video (Video 3), with the number of pixels ranging from 74 to 102. (h) The PA MAP image of the time course of the vessel diameter changes in ketamine-injected rats. (i)–(l) Cross-sectional vessel images at different time points after ketamine injection. (m) The PA B-scan images of ketamine-injected rats were combined in a video to monitor vessel changes (Video 3). (n) The vessel diameter changes were monitored throughout the video (Video 3), with the number of pixels ranging from 98 to 242 (Video 3, MP4, 5.03 MB [URL: https://doi.org/10.1117/1.NPh.9.4.045003.s3).

Figure 7 reports B-scan monitoring of vasoconstriction and vasodilation in groups 3 and 4. Figure 7(a) shows the time course of the blood vessel diameter changes in rats in group 3. Figures 7(b)–7(e) present cross-sections of the blood vessels of rats in group 3 at different time points. Figure 7(f) shows PA B-scan images that were combined in a video to monitor blood vessel changes in group 3 (Video 3). The number of pixels in PA B-scan images of rats in group 3 at various time points is shown in Fig. 7(g) (Video 3), with the number of pixels ranging from 74 to 102. Figure 7(h) shows the time course of blood vessel diameter changes in rats in group 4. Figures 7(i)–7(l) present cross-sections of blood vessels of rats in group 4 at different time points. Figure 7(m) shows PA B-scan images that were combined in a video to monitor blood vessel changes in group 4 (Video 3). The number of pixels in PA B-scan images of rats in group 4 at various time points is shown in Fig. 7(n) (Video 3), with the number of pixels ranging from 98 to 242.

**Fig. 8:**
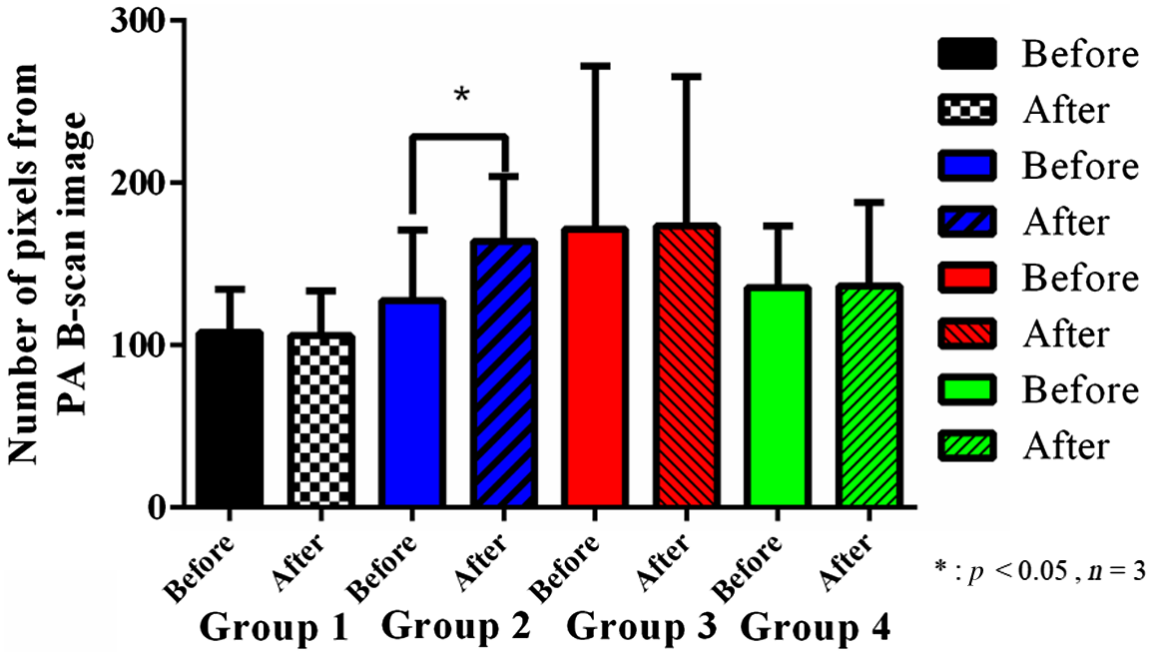
Number of pixels in PA B-scan images of saline-injected and ketamine-injected rats. The black bars indicate the change in group 1. The blue bars indicate the change in group 2. The red bar indicates the change in Group 3. The green bar indicates the change in group 4. The error bars represent the SD. *p<0.05 (paired t-test, n=3). Group 1 represents the changes in the number of pixels in normal rats, with 107.89±26.59  pixels before saline injection and 105.97±27.74  pixels after saline injection, indicating no statistically significant difference [p>0.05 (p=0.2088), paired t-test, n=3]. Group 2 represents the changes in the number of pixels in normal rats, with 127.53±43.48  pixels before ketamine injection and 164.03±40.00  pixels after ketamine injection, demonstrating a statistically significant difference [p<0.05 (p=0.0191), paired t-test, n=3]. The number of pixels in the group 3 images of addicted rats was 196.13±107.76 before saline injection and 192.70±102.19 after saline injection, with no statistically significant difference [p>0.05 (p=0.4414), paired t-test, n=3]. The number of pixels in the group 4 images of addicted rats was 128±42.11  pixels before ketamine injection and 145.67±59.07  pixels after ketamine injection, demonstrating no significant difference [p>0.05 (p=0.2650), paired t-test, n=3]. This finding indicates that ketamine has no obvious effect on the dynamics of cerebral blood vessels in addicted rats, which may be because the addicted rats have adapted to ketamine; thus, the cerebral blood vessels do not undergo any pronounced changes.

The PA B-scan results of the effect of ketamine on the cerebral blood vessels of normal and addicted rats that were obtained with the hmPAI system are shown in Fig. 8. Statistical analysis in GraphPad using paired t-tests showed the changes in the numbers of pixels in PA B-scan images of rats in groups 1, 2, 3, and 4. The numbers of pixels in the PA B-scan image of group 1 before and after saline injection were 107.89±26.59 and 105.97±27.74, respectively [p>0.05 (p=0.2088), paired t-test, n=3]. The numbers of pixels in the PA B-scan image of group 2 before and after ketamine injection were 127.533±43.48 and 164.03±40, respectively, [p<0.05 (p=0.0191), paired t-test; n=3]. The numbers of pixels in the PA B-scan image of group 3 before and after saline injection were 196.13±107.76 and 192.7±102.19, respectively [p>0.05 (p=0.4414), paired t-test, n=3]. The numbers of pixels in the PA B-scan image of group 4 before and after ketamine injection were 128±42.11 and 145.67±59.07, respectively [p>0.05 (p=0.265), paired t-test, n=3].

## 4 Discussion

### 4.1 Using the Developed hmPAI System to Compare Cerebral Hemodynamics in Awake, Freely Moving Normal and Ketamine-Addicted Rats

Our previous experimental study reported that our hmPAI system can be successfully used to detect changes in the CBV and blood vessel diameter in rats in anesthetized and awake rat models in both B-scan and C-scan PA imaging modes.^22^ In the present study, we compared cerebral hemodynamics in response to saline or ketamine injection in freely moving normal rats and prolonged ketamine-exposed rats using the developed hmPAI system. Our findings revealed that the hmPAI system can be used to measure ketamine-induced changes in the CBV and blood vessel diameter in the brain vasculature. In addition, these ketamine-induced changes were significant in normal rats but not significant in prolonged ketamine-exposed rats.

Previous ketamine studies of awake animals were limited to behavioral observations,^26^^,^^27^ electrophysiological recordings,^28^ and other measurements that do not allow for direct visualization of the cerebral dynamics, which can be accomplished by imaging studies. Most previous ketamine imaging studies were conducted in anesthetized animals.^29^^–^^31^ Although these studies enhanced our understanding of physiological processes and the associated neurovascular dynamics, anesthesia had a substantial effect on vascular, electrical, metabolic, and other physiological processes and altered the relationship between neural and vascular components.^32^^–^^38^ For example, anesthesia has been shown to alter how neurotransmitters bind to receptors.^11^ Because ketamine acts as an N-methyl-D-aspartate (NMDA) receptor antagonist, the altered binding caused by anesthesia is a confounding factor in *in vivo* studies on the effect of ketamine on physiological processes. Therefore, compared with the studies conducted on anesthetized animals, our findings in awake, freely moving rats are better related to observations in humans. Caution must be taken when translating data from neuroimaging studies of anesthetized animals to conclusions in awake humans, especially for drug addiction studies.

Additional studies are needed to resolve discrepancies between awake and anesthetized animals. Various investigators have reported differences in neurovascular coupling in awake and anesthetized animals, which could be attributed to different combinations of stimulation parameters (stimulus duration, intensity, frequency, stimulation site, etc.).^39^^–^^41^ These differences can also be attributed to the various anesthesia methods because anesthetic agents have distinct effects on neurovascular coupling and neuronal response patterns.^42^^–^^44^ Thus, the validity of the stimuli used in *in vivo* studies must be carefully considered. A combination of stimulation parameters that can elicit robust responses under anesthesia may not necessarily be relevant in the awake state.

Our findings are the first to characterize the modulatory effects of ketamine-induced hemodynamic changes in awake rats. We found that the blood vessel diameter changed in response to ketamine stimulation in normal rats, whereas the blood vessels of rats subjected to prolonged ketamine self-administration did not change significantly in response to ketamine stimulation. Several previous functional MRI studies of ketamine injection in awake subjects have also shown cerebral activation induced by ketamine in nonaddicted subjects.^45^^,^^46^ In a PET study that compared anesthetic and subanesthetic doses of S-ketamine, both anesthetic and subanesthetic doses were found to induce significant increases in whole-brain cerebral blood flow in human subjects.^47^ The subanesthetic ketamine dose used in the present study corresponds to doses used to treat depressive disorder. We found that subanesthetic doses of ketamine increased blood vessel diameter and CBV in normal rats but did not affect the blood vessel diameter and CBV in rats subjected to prolonged ketamine self-administration. After prolonged ketamine self-administration, the rats appeared to develop a tolerance toward the dilative effect of ketamine in the cerebral vasculature. In imaging studies of anesthetized animals,^31^^,^^47^ cerebral activation in animals that were chronically injected with ketamine was lower than that in normal animals, which corresponds to our results.

### 4.2 Future Applications of the Developed hmPAI System for Studying Pharmacological Effects in Awake, Freely Moving Animals

The developed hmPAI system uses PA effects to detect cerebral activation. PA imaging cannot be used to directly measure neuronal and glial activation;^9^ instead, PA imaging detects changes in hemodynamic activities resulting from neuronal and glial activation. This process assumes that blood flow and neural activities are directly related. Although this assumption is true under normal conditions, recent studies have noted that neurovascular coupling may be abnormal in certain diseases^48^^,^^49^ or under the influence of certain drugs, such as ketamine, which was used in this study. This finding would limit the direct application of PA signals in neuroimaging. However, Wang et al. recently reported a new PA solution that allows users to simultaneously image cerebrovascular anatomy, the total concentration and hemoglobin oxygen saturation, and blood flow in awake mice.^50^ By combining these hemodynamic parameters, PA technology allows the cerebral metabolic rate of oxygen (${\mathrm{CMRO}}_{2}$) to be determined, which might open a new avenue for directly detecting metabolic rates.^51^ Whole-brain direct imaging of neuronal activity in real time has faced significant challenges. Gottschalk et al.^52^ used mice that were genetically engineered to express the calcium indicator GCaMP6f to demonstrate *ex vivo* and noninvasive *in vivo* functional PA neuroimaging. This approach enables rapid, high-resolution mapping of whole-brain neuronal activity while simultaneously monitoring hemodynamic changes.

In addition to PA neuroimaging techniques, significant efforts have been dedicated to developing preclinical imaging techniques that can resolve neurovascular communications, especially in awake animals. Our results show that the proposed hmPAI system can be used to image awake, freely behaving rodents; thus, our novel system is an optimal tool for understanding hemodynamic functions. Data on cerebral hemodynamics in response to functional stimulation is more realistic when the animal is awake and freely moving.^51^ Additionally, various details, such as vasodilatory responses, the intensity, and latency/recovery times, allow for a more in-depth understanding of hemodynamic functions.

Although standard optical imaging methods, including LSCI^53^^–^^55^ and TPM,^55^^,^^56^ can be used for neurovascular imaging of awake and freely moving animals, PA imaging has several advantages over standard optical imaging methods. LSCI can be used to image large areas, provides blood flow information, and achieves a spatial resolution of up to 10  μm.^57^ However, LSCI can only be used to image superficial blood vessels and does not provide 3D images. Although the hmPAI system was used to image the SSS, a superficial blood vessel, in this study, this system can be used to image deeper cerebral regions (up to 10 mm below the transducer surface)^15^ and produce C-scan images. TPM has a higher spatial resolution and can be used to image individual cells. However, TPM has several disadvantages, including a smaller field of view (FOV) and the need for fluorescence labels. In contrast, PA imaging can be used to observe larger FOVs and images blood vessels based on intrinsic contrast. Other groups have also proposed PA systems for imaging awake animals. In traditional, bulkier PA systems, animals are fixed in a head restraint on a treadmill to allow for some movement.^51^^,^^58^ This type of setup prevents issues such as motion artifacts and large system weights, ensuring that complex lens and mirror systems can be used for high-resolution imaging. However, the behavior of the restrained animal does not completely reflect normal behavior. Thus, wearable systems with much smaller weights (1.8 to 8 g) than conventional PA systems have been designed.^59^^–^^62^ The use of microelectromechanical system (MEMS) scanning reduces the system size by eliminating the need for bulky linear motors when performing B- or C-scans. Transparent ultrasound transducers (TUTs) have also been used in wearable PA systems.^59^^–^^61^ TUTs are thin piezoelectric films that can be used as cranial windows. The use of TUTs not only decreases the overall size of the system but also ensures that bone regrowth does not obscure the FOV during longitudinal imaging studies. The weight of wearable PA systems for rats can reach as low as 8 g,^60^ whereas the weight of the hmPAI system is 58.7 g.^61^^,^^63^ The axial resolutions of existing wearable PA systems range from 105 to 202.5  μm,^61^^,^^64^ whereas the axial resolution of the hmPAI system is 225  μm.^22^ However, the main goal of the hmPAI system was to design a simple setup with lower costs, which leads to limitations, such as an increased weight and lower resolution.

Our system has several limitations. The developed hmPAI system is currently limited to a single imaging plane and relies on a scanning motor to move the probe in B- and C-scan imaging. Thus, the hmPAI system can be improved further using miniature 2D matrix array transducers to increase the FOV^65^ or MEMS technology-based scanning systems^66^ to increase the imaging speed. These improvements would allow us to use larger animal models, such as rabbits, or to perform whole-brain imaging. However, these changes would also limit the large amount of real-time data collected and the number of reconstructed voxels that need to be stored and processed for real-time 3D imaging datasets. A graphics processing unit can be used to accelerate real-time 3D reconstructions.^67^ Another limitation of the hmPAI system is the bulky size of the laser system. Recent studies have utilized laser diodes and light-emitting diodes to improve the portability of PA systems.^68^ However, the lower output energy of these systems often results in a lower signal-to-noise ratio, and coded excitation methods or signal processing methods such as empirical mode decomposition, Wiener deconvolution, and wavelet-based methods are needed to improve the system resolution.^69^^,^^70^

In this study, we demonstrated that the developed hmPAI system can be used to monitor hemodynamic changes in response to ketamine stimulation and that differences between normal and ketamine-addicted animals can be detected. Future applications can combine behavioral studies with imaging to simultaneously study neural function and behavior in addicted animals. Longitudinal studies can also be used to monitor the long-term effects of addiction. Finally, the studies in ketamine-addicted animals can be extended to addiction studies for amphetamines and other controlled substances.

## 5 Conclusion

We developed a hmPAI system that includes a detachable fiber bundle-based illumination system and a single transducer ultrasound platform that can be used to successfully examine cerebral hemodynamics in awake, ketamine-addicted rats. Our method utilizes fiber bundle-based dark-field illumination and a 2D scanning mechanism with four linear servo motors. Our hmPAI system monitored ketamine-induced changes in cerebral blood vessel diameters and blood volume *in vivo*. After saline injection in normal rats, the change in the diameter of the cerebral blood vessels in A-line signals relative to the baseline was 93.71±10.47%, and the change in the diameter of the cerebral blood vessels in normal rats after ketamine injection was 120.42±4.89% relative to the baseline. Statistical analyses indicated that this change was significant (p<0.05). There was no significant change in ketamine-addicted rats that were treated acutely with either saline or ketamine. The PA B-scan results of the number of pixels as an indicator of the CBV were similar. Ketamine injection in normal rats led to a significant increase in the number of pixels, from 127.53±43.48 to 164.03±40.00 (p<0.05). In addicted rats, however, ketamine injection did not induce a significant change in the CBV. There was also no significant change in control rats (saline injection in normal and addicted rats). We are the first group to use a wearable hmPAI system to monitor changes in cerebral blood vessels and blood volume in response to ketamine. The hmPAI system can be used to study different experimental models, such as the ketamine addiction model in awake, freely moving rats, in future work. We can accurately monitor interesting cerebral regions, and the developed system could be used in functional brain research. Overall, the developed hmPAI system can complement existing optical imaging techniques and has the potential to be a useful tool for different experimental models in brain research.
